# Disruption of the Interfacial Membrane Leads to *Magnaporthe oryzae* Effector Re-location and Lifestyle Switch During Rice Blast Disease

**DOI:** 10.3389/fcell.2021.681734

**Published:** 2021-06-17

**Authors:** Kiersun Jones, Jie Zhu, Cory B. Jenkinson, Dong Won Kim, Mariel A. Pfeifer, Chang Hyun Khang

**Affiliations:** Department of Plant Biology, University of Georgia, Athens, GA, United States

**Keywords:** cell death, effector proteins, hemibiotrophy, host-pathogen interface, live-cell imaging, plant-fungal interactions, plasmodesmata, vacuoles

## Abstract

To cause the devastating rice blast disease, the hemibiotrophic fungus *Magnaporthe oryzae* produces invasive hyphae (IH) that are enclosed in a plant-derived interfacial membrane, known as the extra-invasive hyphal membrane (EIHM), in living rice cells. Little is known about when the EIHM is disrupted and how the disruption contributes to blast disease. Here we show that the disruption of the EIHM correlates with the hyphal growth stage in first-invaded susceptible rice cells. Our approach utilized GFP that was secreted from IH as an EIHM integrity reporter. Secreted GFP (sec-GFP) accumulated in the EIHM compartment but appeared in the host cytoplasm when the integrity of the EIHM was compromised. Live-cell imaging coupled with sec-GFP and various fluorescent reporters revealed that the loss of EIHM integrity preceded shrinkage and eventual rupture of the rice vacuole. The vacuole rupture coincided with host cell death, which was limited to the invaded cell with presumed closure of plasmodesmata. We report that EIHM disruption and host cell death are landmarks that delineate three distinct infection phases (early biotrophic, late biotrophic, and transient necrotrophic phases) within the first-invaded cell before reestablishment of biotrophy in second-invaded cells. *M. oryzae* effectors exhibited infection phase-specific localizations, including entry of the apoplastic effector Bas4 into the host cytoplasm through the disrupted EIHM during the late biotrophic phase. Understanding how infection phase-specific cellular dynamics are regulated and linked to host susceptibility will offer potential targets that can be exploited to control blast disease.

## Introduction

Plants grow under constant threat of attack by diverse pathogens, ranging from obligate biotrophs that require living host cells to necrotrophs that benefit from host cell death. One group of pathogenic fungi, known as hemibiotrophs, suppress host cell death during an initial biotrophic phase but later induce host cell death during a necrotrophic phase (Perfect and Green, 2001). A hallmark of biotrophic growth is the encapsulation of fungal structures, such as intracellular invasive hyphae (IH), within a host-derived membrane that separates the pathogen from the host cytoplasm. The host-derived membrane serves as a critical interface for maintaining biotrophy, permitting the pathogen to evade host recognition while acquiring nutrients from the host (Perfect and Green, 2001; Bozkurt et al., 2015). Our understanding of how these plant-pathogen interfaces initially form and persist is limited, particularly in hemibiotrophs, which transition from biotrophic to necrotrophic growth phases (Perfect and Green, 2001).

**FIGURE 1:**
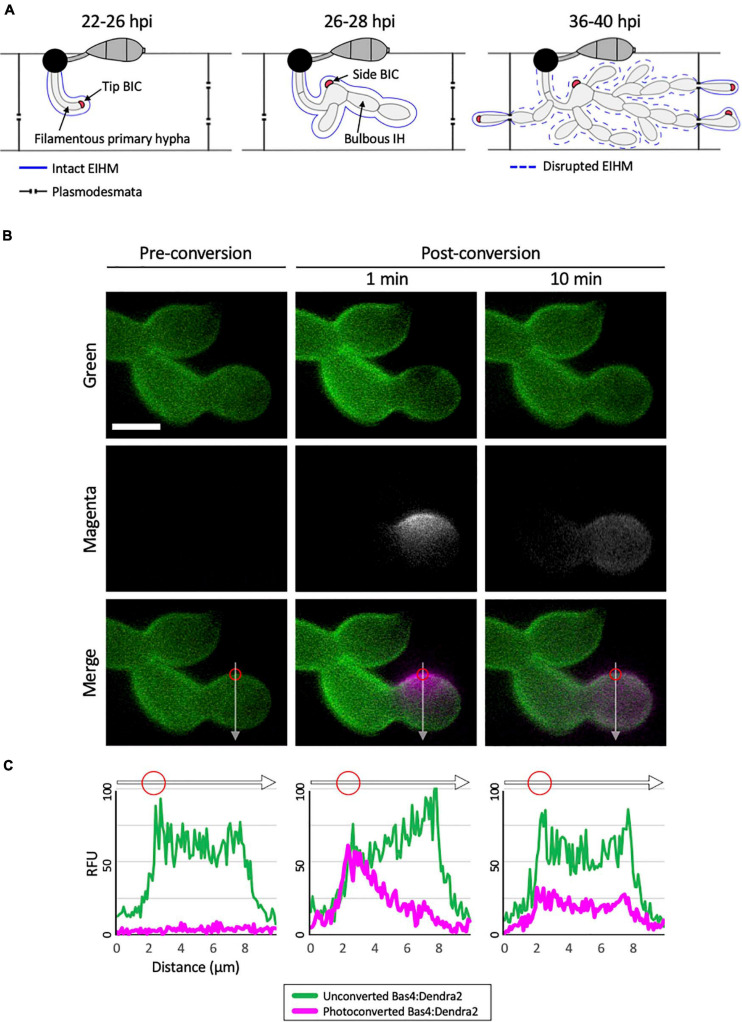
Bas4 is freely diffusible inside the EIHMx. **(A)** Schematic diagram summarizing the invasion of first and second rice cells by *M. oryzae* invasive hyphae (IH). At 22–26 h post inoculation (hpi), a filamentous primary hypha grows in the first-invaded host cell, where it is surrounded by an intact EIHM. Apoplastic effectors secreted by IH are retained within the EIHMx. In contrast, cytoplasmic effectors enter the host cytoplasm and show preferential accumulation at the tip BIC located at the apex of the primary hypha. At 26–28 hpi, the filamentous primary hypha switches to depolarized, asymmetric growth, leaving the BIC subapically associated with the first bulbous IH cell, becoming a side BIC. Polarized growth resumes from the BIC-associated cell, producing bulbous IH. The EIHM remains intact. At 36–40 hpi, the EIHM in the first-invaded host cell is disrupted, and IH invade neighboring host cells. Every IH that invades an adjacent rice cell is surrounded by a new EIHM and associated with a new BIC. **(B)** IH of *M. oryzae* CKF1737 expressing EIHMx-localized effector Bas4 fused to the green-to-red photoconvertible fluorescent protein Dendra2, invading a rice cell at 29 hpi. Shown are single plane confocal images of separate fluorescence (top and middle panels) and merged fluorescence (bottom panels). Left: Before photoconversion, green Bas4:Dendra2 fluorescence localized throughout the EIHMx. Middle: 1 min after selective photoconversion (region indicated by the red circle), red Bas4:Dendra2 fluorescence (magenta and pseudo-colored white) diffused into the surrounding EIHMx. Right: 10 min after photoconversion, the red Bas4:Dendra2 further diffused. White arrows indicate the locations of fluorescence intensity linescans shown in panel **(C)**. Bar = 5 μm. **(C)** Linescans showing the relative fluorescence intensity between unconverted Bas4:Dendra2 (green) and photoconverted Bas4:Dendra2 (magenta), corresponding to the location of the white lines in panel **(B)**. Red circles show the photoconverted region in panel **(B)**. Units are relative fluorescence units (RFU; y-axis) and distance in μm (x-axis).

*Magnaporthe oryzae* is a hemibiotrophic ascomycete responsible for the economically devastating blast disease on rice, wheat and other crops (Khang and Valent, 2010; Pennisi, 2010; Cruz and Valent, 2017). The fungus successively invades living rice cells (biotrophy) before switching to destructive growth associated with macroscopic lesion development and conidiation (necrotrophy) several days after inoculation (Kankanala et al., 2007; Khang and Valent, 2010). The cellular features and events of *M. oryzae* during the early biotrophic invasion have been documented (Figure 1A; Koga et al., 2004; Kankanala et al., 2007; Mochizuki et al., 2015; Jones et al., 2016a,b; Jenkinson et al., 2017; Shipman et al., 2017; Pfeifer and Khang, 2018, 2021; Pfeifer et al., 2019). On the rice leaf surface the fungus forms an appressorium, which produces a narrow penetration peg to breach an epidermal rice cell. The penetration peg expands to form a filamentous primary invasive hypha, which subsequently differentiates into bulbous IH, growing within the first-invaded host cell for 8–12 h before moving into adjacent cells using IH pegs that co-opt plasmodesmata (PD) of rice cells (Kankanala et al., 2007; Sakulkoo et al., 2018). Importantly, biotrophic IH are surrounded by a host-derived tight-fitting extra-invasive hyphal membrane (EIHM) (Kankanala et al., 2007; Yi and Valent, 2013; Kouzai et al., 2014; Mochizuki et al., 2015). Several studies noted that the EIHM could lose integrity during IH growth in first-invaded rice cells (Mosquera et al., 2009; Khang et al., 2010; Mochizuki et al., 2015; Jones et al., 2016b). This loss of EIHM integrity during *M. oryzae* invasion of rice is a stark contrast to biotrophic pathogens where the integrity of the pathogen-plant membrane interface is maintained. In addition, first-invaded rice cells exhibit shrinkage and rupture of the central vacuole with loss of rice cell viability around the time IH of *M. oryzae* move into adjacent living cells (Kankanala et al., 2007; Mochizuki et al., 2015; Jones et al., 2016b; Sakulkoo et al., 2018). However, the precise sequence of these cytological events has not been clearly defined.

To facilitate infection of the host, pathogens secrete effector proteins, some of which modulate host immune responses and cell death, depending on infection stage (Kleemann et al., 2012; Giraldo and Valent, 2013; Toruño et al., 2016; Lanver et al., 2017; Zhu et al., 2021). Live-cell imaging of *M. oryzae* strains expressing fluorescently-tagged effectors has shown differential subcellular localization of effectors after they are secreted from IH *via* two distinct protein secretion pathways (Giraldo et al., 2013). Apoplastic effectors (i.e., Bas4, Bas113, and Slp1) are retained in the extrainvasive hyphal matrix (EIHMx), which is the sealed apoplastic compartment formed between the EIHM and IH cell wall (Kankanala et al., 2007; Mosquera et al., 2009; Mentlak et al., 2012; Yi and Valent, 2013). In contrast, cytoplasmic effectors (i.e., Pwl2, Bas1, Bas107, and AvrPiz-t) preferentially accumulate in the biotrophic interfacial complex (BIC), which is a plant-derived structure that is enclosed by the EIHM (Khang et al., 2010; Park et al., 2012; Giraldo et al., 2013). BICs have been hypothesized to be the site of effector translocation across the EIHM into the host cytoplasm and undergo a two-stage developmental process (Khang et al., 2010; Giraldo and Valent, 2013; Giraldo et al., 2013).

The two-stage development of BICs, from tip- to side-BIC, occurs in conjunction with IH differentiation from filamentous to bulbous in living rice cells (Figure 1A; Khang et al., 2010; Shipman et al., 2017). Only a single BIC is present in each first-invaded cell, whereas multiple BICs can be present in subsequently invaded adjacent cells, one associated with each IH entering them (Figure 1A). Cell-to-cell movement of effectors during the early infection stage indicated symplastic continuity of rice cells is maintained *via* open PD between the first-invaded cell and uninvaded adjacent cells (Kankanala et al., 2007; Khang et al., 2010). However, live-cell imaging of *M. oryzae*-invaded rice cells coupled with fluorescein diacetate (FDA) staining at a later infection stage showed that PD permeability was reduced during host vacuole shrinkage and after vacuole rupture in the first-invaded cell (Jones et al., 2016b). This reduction in PD permeability is consistent with reported increase in callose deposition in pit fields, the sites of PD, at onset of cell death in the first-invaded rice cell (Sakulkoo et al., 2018). Thus, the reported localization of effectors is likely dependent on the integrity of the EIHM and other host membranes, along with the permeability of PD during rice cell infection. Despite advances in our understanding of rice blast infection dynamics, little is known as to how these complex plant-pathogen cellular events are coordinated.

In this study, we used live-cell imaging of susceptible rice cells invaded by *M. oryzae* transformants expressing various fluorescent reporters to investigate infection development in first- and second-invaded cells. We show that EIHM disruption occurred in the first-invaded cell, contingent on IH growth stage, followed by shrinkage and eventual rupture of the rice vacuole before IH spread into adjacent cells. Vacuole rupture coincided with host cell death, which occurred in a contained manner with presumed closure of PD. We demonstrate that *M. oryzae* undergoes three distinct infection phases in the first-invaded cell before reestablishing biotrophy in the second-invaded cells. *M. oryzae* effectors exhibited infection phase-specific localization. Understanding how the infection phase-specific cellular dynamics are regulated and linked to host susceptibility will offer potential targets that we can exploit to control blast disease.

## Results

### Secreted Proteins Are Mobile in the EIHMx

To investigate the nature of the rice-*M. oryzae* interface, we generated an *M. oryzae* transformant expressing Bas4 as a translational fusion to the photoconvertible fluorescent protein Dendra2, which can be irreversibly changed from green to red fluorescence upon irradiation with UV light (Gurskaya et al., 2006). Bas4 is an *M. oryzae* effector protein that contains a N-terminal signal peptide (SP; 21 amino acids) which mediates the secretion of the leaderless Bas4 (81 amino acids) into the EIHMx (Mosquera et al., 2009; Khang et al., 2010; Giraldo et al., 2013). During invasion of rice cells the transformant showed bright green fluorescence around IH, indicating that Bas4:Dendra2 was indeed secreted into the EIHMx. This localization pattern was consistent with patterns that have been observed for Bas4 fused to other fluorescent proteins, such as EGFP or mCherry (Mosquera et al., 2009; Khang et al., 2010; Mochizuki et al., 2015). To investigate the mobility of Bas4:Dendra2 in the EIHMx, we selectively photoconverted a small region of Bas4:Dendra2 and then monitored dynamics of both the converted red and the unconverted green fluorescence (Figure 1B). Photoconverted Bas4:Dendra2 progressively diffused into the surrounding EIHMx over the next several minutes, meanwhile unconverted Bas4:Dendra2 diffused into the photoconverted region (Figures 1B,C). These results indicated that secreted proteins are diffusible in the EIHMx.

### Secreted GFP as a Reporter for EIHM Integrity and Other Host Cellular Dynamics

Considering the mobility of secreted Bas4:Dendra2 within the EIHMx (Figure 1B), we reasoned that secreted GFP (sec-GFP) could be used to monitor the integrity of the EIHM. In the case of a completely intact EIHM, sec-GFP would be retained exclusively within the EIHMx; conversely, if EIHM integrity is compromised, sec-GFP would spill from the EIHMx into the rice cell lumen. To test this, we used an *M. oryzae* strain expressing a fusion of GFP with the Bas4 signal peptide coupled with the lipophilic dye FM4-64 staining. FM4-64 was previously shown to label fungal membranes, notably at septa, only when the EIHM integrity was compromised (Kankanala et al., 2007). Consistent with this, we found that FM4-64 was visible at fungal septa only when sec-GFP appeared in the rice cell lumen (see Supplementary Figure 1). These results demonstrated the utility of sec-GFP localization to reveal EIHM integrity.

**FIGURE 2:**
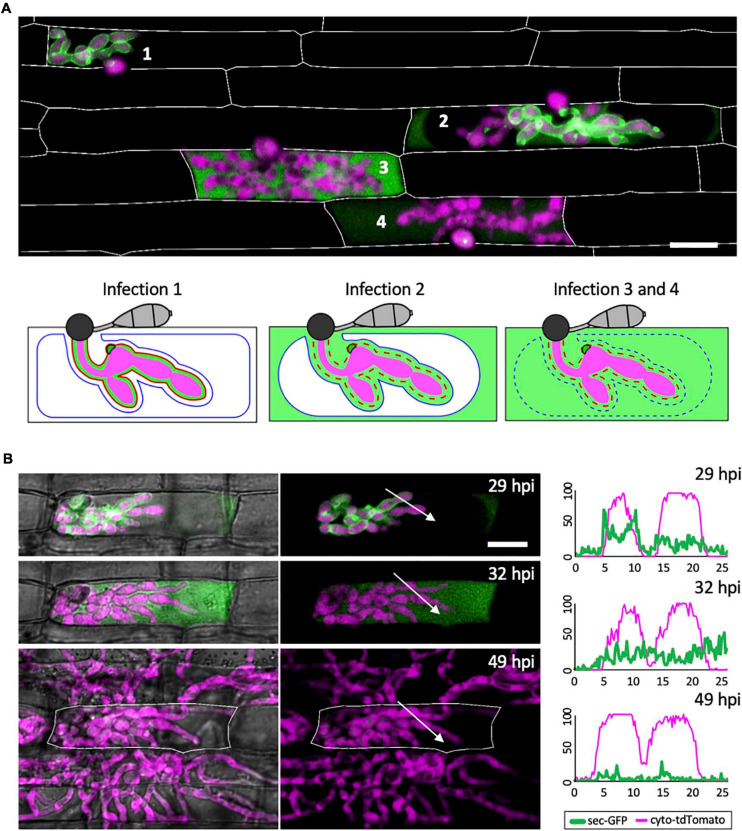
The EIHM loses integrity during invasion of the first host cell. **(A)** A merged fluorescence projection of *M. oryzae* CKF1996, expressing sec-GFP (green) and cytoplasmic tdTomato (magenta) in first-invaded rice cells at 32 hpi. Infections are representative of the patterns observed for sec-GFP localization: retention within the EIHMx (infection 1), spilled into the rice cytoplasm with exclusion from the vacuole (infection 2), and spilled homogenously into the rice cell lumen with a ruptured vacuole (infection 3 and 4). Rice cell walls are denoted by white outlines. The same three sec-GFP patterns are schematically illustrated with the addition of the EIHM membrane (red line) and the vacuole membrane (blue line). Disrupted membranes are indicated by a dotted line. Bar = 20 μm. **(B)** Shown are merged fluorescence and bright-field (left) and merged fluorescence alone (right) of a time-lapsed CKF1996 infection from 29 to 49 hpi. At 29 hpi, sec-GFP fluorescence (green) was partially spilled from the EIHMx into the rice cytoplasm and excluded from the vacuole. Three hours later, all sec-GFP fluorescence was homogenously distributed throughout the rice cell. After another 17 h, viable IH (magenta) had exited the first-invaded host cell (white outline) and had successfully invaded and colonized multiple adjacent rice cells. White arrows denote the locations used for generating the fluorescence intensity linescans. Bar = 20 μm. The linescans measure the relative fluorescence intensity of sec-GFP (green) and cytoplasmic tdTomato (magenta). At 29 hpi, two neighboring hyphae have different localization patterns of sec-GFP; one hypha is outlined by sec-GFP fluorescence (represented by the two green fluorescence peaks) while the other hypha does not show sec-GFP outlining. At 32 hpi, the same two hyphae both now lacked sec-GFP outlining. At 49 hpi, the same IH did not show significant sec-GFP fluorescence. Units are relative fluorescence units (RFU; y-axis) and distance in μm (x-axis).

Three distinct patterns of sec-GFP localization were identified by live-cell imaging of first-invaded rice cells infected with *M. oryzae* strain CKF1996, expressing sec-GFP together with cytoplasmic tdTomato (*n* > 100). The first pattern was sec-GFP exclusively localized in the EIHMx, indicating an intact EIHM (Figure 2A; infection 1). The second pattern was sec-GFP localized in the rice cytoplasm excluded from the shrunken vacuole (Figure 2A; infection 2; Supplementary Figure 2). The third pattern was homogenous distribution of sec-GFP throughout the rice cell with the collapsed vacuole (Figure 2A; infection 3 and 4). These results were consistent with recently reported shrinkage and collapse of the central vacuole in *M. oryzae*-invaded rice cells (Mochizuki et al., 2015; Jones et al., 2016b). Interestingly, spilled sec-GFP did not diffuse into neighboring rice cells (Figure 2; see Supplementary Figures 1, 2), suggesting PD were closed. Together, these results showed that sec-GFP localization provides a robust assay for the integrity of the EIHM as well as the state of the host vacuole and PD permeability after EIHM disruption.

### Fungal Colonization Continues After EIHM Disruption

Not all infections result in successful colonization of the host even when it is a susceptible interaction (Heath et al., 1990). We therefore considered that EIHM disruption could be associated with failed infection. Thus, we tested if IH growth becomes arrested after EIHM disruption in the first-invaded rice cell. Time-lapse imaging of rice cells invaded by the *M. oryzae* strain CKF1996 (sec-GFP and cytoplasmic tdTomato) showed that IH continued to colonize host cells after the EIHM was disrupted during invasion of the first host cell (Figure 2B; *n* = 15). The growth of IH in the time-lapsed infections was consistent with freshly prepared control infections that were not subject to any potential imaging-related stress (data not shown). Therefore, we concluded that disruption of the EIHM during invasion of the first cell is characteristic of successful colonization of rice by *M. oryzae.* These time-lapse imaging results also revealed that sec-GFP first spilled into the rice cytoplasm with exclusion from the shrinking central vacuole, followed by homogenous distribution throughout the rice cell upon vacuole rupture and that IH subsequently spread into adjacent host cells (Figure 2B). This sequence of events was consistent with previous reports (Mochizuki et al., 2015; Jones et al., 2016b), however, in addition it revealed that the EIHM disrupts before the vacuole ruptures.

### EIHM Disruption Process

**FIGURE 3:**
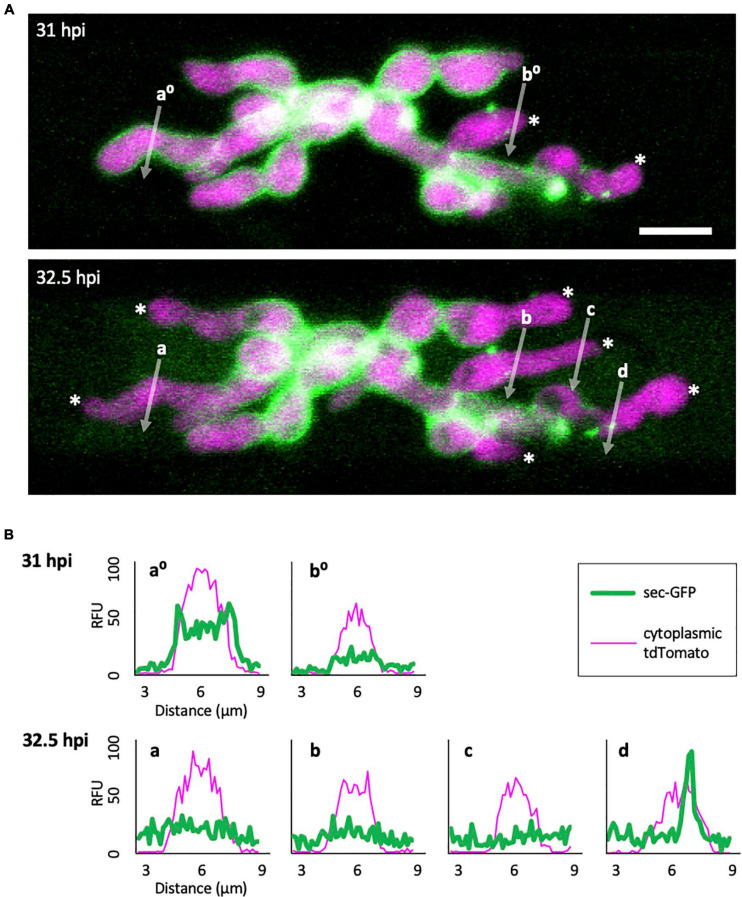
Sec-GFP localization changes during the process of EIHM disruption. **(A)** *M. oryzae* CKF1996 expressing sec-GFP (green) and cytoplasmic tdTomato (magenta) invading a rice cell. Shown are merged fluorescence projections of informative focal planes from the same infection site at 31 (top) and 32.5 hpi (bottom). At 31 hpi sec-GFP localized in the host cytoplasm (not shown in the field of view) and outlined most IH (IH without asterisks and linescan a^0^). Two IH had lost sec-GFP outlining (white asterisks and linescan b^0^). The same infection 1.5 h later had increased sec-GFP accumulation in the host cell (now visible in the field of view) and loss of sec-GFP outline from additional IH (increase from 2 to 6 white asterisks). White letters and arrows denote the locations used to generate the fluorescence intensity linescans shown in panel **(B)**. Bar = 10 μm. **(B)** Linescans showing the relative fluorescence intensities (RFU; y-axis, distance in μm; x-axes) of sec-GFP (green) and cytoplasmic tdTomato (magenta) from panel **(A),** highlighting features of sec-GFP fluorescence localization changes during the process of EIHM disruption. Linescans at 31 hpi show an IH with a sec-GFP outline (a^0^) and an IH without a sec-GFP outline (b^0^). Linescans generated from the same locations at 32.5 hpi show new loss of sec-GFP outline (a) and maintained absence of sec-GFP outlining after initial loss (b). Additional linescans show: new IH growth after sec-GFP outline loss without accumulation of new sec-GFP outline (c), and a sec-GFP puncta associated with outline loss (d). Note that linescans generated at 32.5 hpi clearly show sec-GFP fluorescence spilled into the rice cell lumen (green fluorescence not associated with IH).

To gain insight into how the EIHM is disrupted, we observed the early stage of sec-GFP spilling in first-invaded cells (*n* = 18) and identified four features associated with EIHM disruption: (1) the initial loss of sec-GFP from the EIHMx appeared to occur near the tips of growing IH (Figure 3; asterisks, linescan a^0^ and a), indicating the initial EIHM disruption at the expanding hyphal region, (2) sec-GFP outline disappeared from some IH while others retained sec-GFP outline (Figure 3A; increase in the number of IH denoted by white asterisks), suggesting that EIHM disruption occurred separately at different locations and was not globally executed, (3) IH that had lost sec-GFP outline did not recover outline, and IH continued to grow without accumulation of new sec-GFP outlining (Figure 3; linescans b^0^, b, and c), indicating that disruption of the EIHM was permanent, and (4) sec-GFP frequently appeared in a punctate pattern at the surface of IH associated with the loss of sec-GFP outline (Figure 3; white arrowheads, linescan d). These puncta varied significantly in terms of quantity, intensity, and duration. The nature of these sec-GFP puncta remains to be determined.

### The Occurrence of EIHM Disruption Increases With IH Growth Stage

**FIGURE 4:**
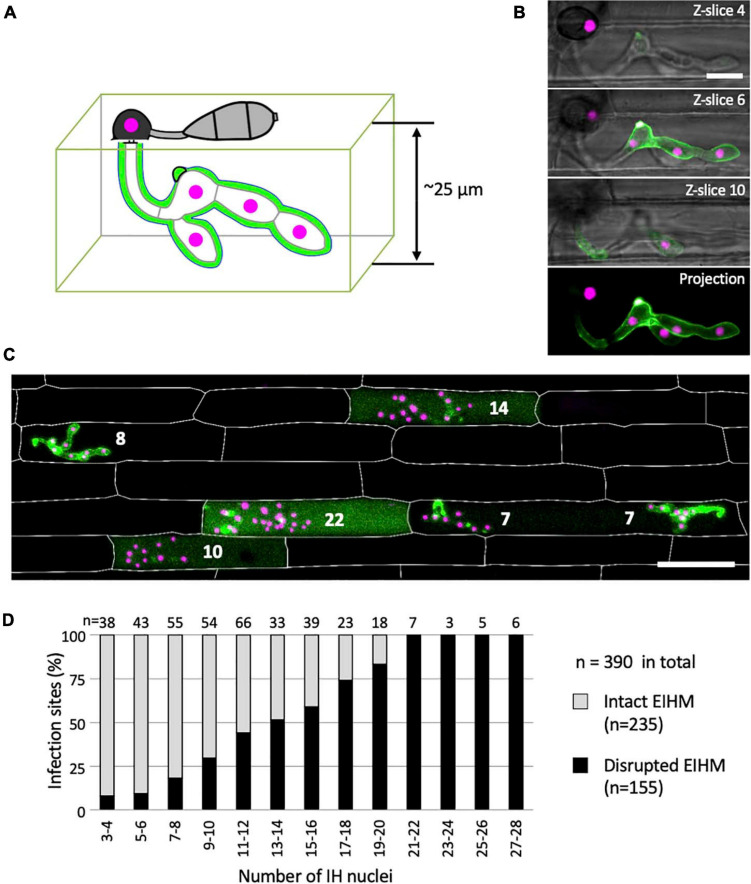
The occurrence of EIHM disruption increases proportionally with nuclear stage. **(A)** Schematic diagram three-dimensionally depicting an epidermal rice cell invaded by *M. oryzae* expressing sec-GFP (green) and nuclear tdTomato (magenta). **(B,C)** Confocal images of rice cells invaded by *M. oryzae* CKF2187 expressing sec-GFP (green) and nuclear tdTomato (magenta). **(B)** Images of the same infection taken at different focal planes. The top three panels show merged fluorescence and bright-field from individual focal planes (indicated in the top right corner), while the last panel shows a merged fluorescence projection of all focal planes; 12 z-slices in total spanning 24 μm over the z-axis. Note that all five fungal nuclei are fully visible only in the projection view. Bar = 10 μm. **(C)** A merged fluorescence projection of infected rice cells at 30 hpi showing different patterns of sec-GFP localization at different IH growth stages determined by the nuclear number for each infection (white numbers). The infection to the far left shows EIHMx-localized sec-GFP (intact EIHM), while the other infections show host-localized sec-GFP (disrupted EIHM). Note the infected rice cell at the bottom right was invaded by two separate appressoria. Rice cell walls are indicated by white outlines. Bar = 50 μm. **(D)** A plot of the frequency of EIHM disruption according to fungal nuclear number for 390 infections of *M. oryzae* CKF2187 in the first-invaded host cell between 28 and 33 hpi. Sec-GFP localization patterns revealed the distribution of intact (*n* = 235) and disrupted (*n* = 155) EIHMs (y-axis) at each group of two nuclear stages (x-axis).

We determined the relationship between EIHM disruption and IH growth stage by analyzing 390 rice cells infected with *M. oryzae* strain CKF2187, expressing sec-GFP and a translational fusion of tdTomato to histone H1 (H1:tdTomato; Figure 4). The growth stage was determined by counting H1:tdTomato-tagged nuclei because each IH cell contains one nucleus. We implemented an empirically-derived image analysis method to increase the sensitivity to low intensity sec-GFP fluorescence in the host cytoplasm so that infections at the early stage of sec-GFP spill were correctly scored (see Supplementary Figure 3). We found that of 390 infections, 235 had an intact EIHM, and 155 had a disrupted EIHM (Figure 4D). Most the 155 infections with a disrupted EIHM showed sec-GFP spilled into either the cytoplasm or homogenously with the disrupted vacuole. However, about ∼7% (*n* = 11 of 155) showed alternative patterns of localization, such as brighter accumulation of sec-GFP in the vacuole (see Supplementary Figure 4). We found that EIHM disruption occurred as early as the 3–4 nuclear stage, although at a low frequency (Figure 4D; *n* = 3 of 38). By the 13–14 nuclear stage EIHM disruption frequency reached about 50% (Figure 4D; *n* = 17 out of 33). All infections at the 21 or more nuclear stage exhibited EIHM disruption (Figure 4D; *n* = 21 of 21). These results showed that the occurrence of EIHM disruption in the first-invaded cell increased proportionally with IH growth as the fungus occupied increased space within the rice cell.

### Death of the First-Invaded Rice Cell Coincides With Vacuole Rupture

**FIGURE 5:**
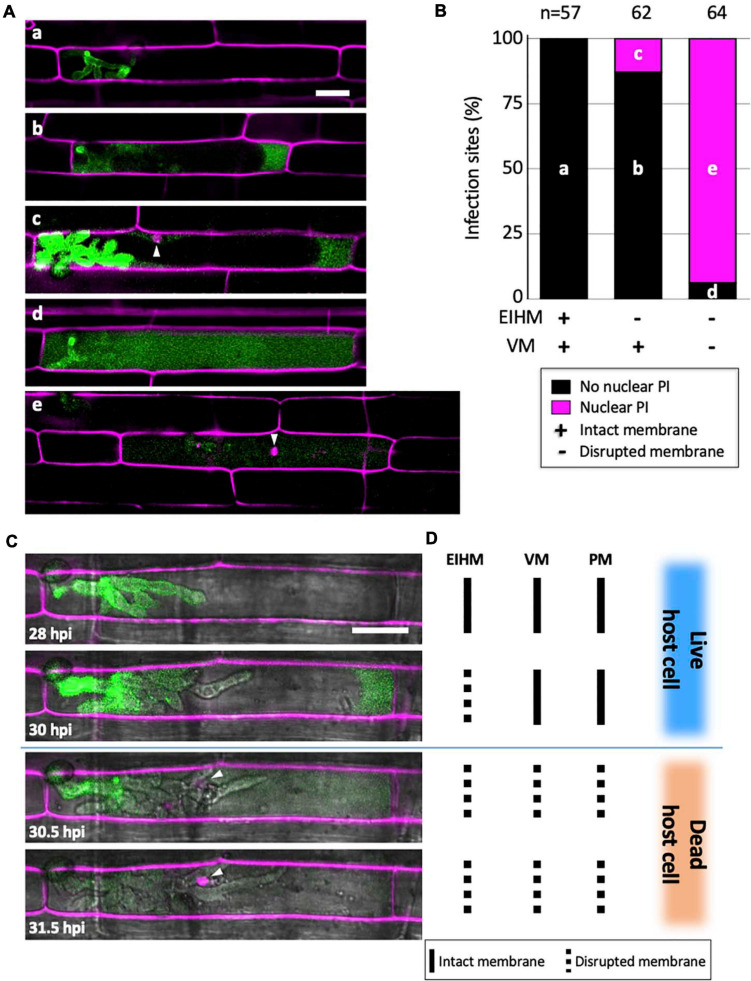
Vacuole rupture indicates host cell death. **(A,C)** Rice cells invaded by *M. oryzae* CKF315 (sec-GFP; green) and stained with propidium iodide (PI; magenta). Shown are single plane confocal images of merged fluorescence **(A)** and merged bright-field and fluorescence **(C)**. White arrowheads indicate rice nuclei stained with PI. Bars = 20 μm. **(A)** Representative images from the 183 infections from 29 to 34 hpi showing the five sec-GFP and PI localization patterns observed: (a) EIHMx-exclusive sec-GFP without nuclear PI stain, (b) sec-GFP spilled into the rice cytoplasm without nuclear PI stain, (c) same as (b) but with nuclear PI stain, (d) sec-GFP homogenized throughout the host cell lumen without nuclear PI stain, (e) same as (d) but with nuclear PI stain. **(B)** Graph showing the distribution of the five sec-GFP and PI fluorescence patterns shown in panel **(A)** for all 183 infections. Black bars and magenta bars represent infections without nuclear PI staining and with nuclear PI staining, respectively. **(C)** Time-lapse series of a PI-stained CKF315 infection from 21 to 31 hpi showing the typical progression of sec-GFP and PI fluorescence localization changes, consistent with quantitative results in panel **(B)**. Note that appearance of host nuclear PI fluorescence coincided with homogenization of host-localized sec-GFP fluorescence. **(D)** Schematic diagram summarizing the states of host membranes, corresponding to the infection shown in panel **(C)**. EIHM, extrainvasive hyphal membrane; VM, host vacuole membrane; PM, host plasma membrane.

To determine when viability is lost in the first-invaded cell, we infected rice cells with *M. oryzae* strain CKF315 expressing sec-GFP and then stained with propidium iodide (PI) just before microscopy. PI staining was previously used to identify dead rice cells during blast invasion because it indiscriminately labels plant cell walls but only labels nuclei when the plasma membrane (PM) is permeabilized, thereby indicating a dead or dying cell (Jones et al., 2016b). We found that infections with an intact EIHM did not show nuclear PI labeling, indicating invaded rice cells were viable as expected (Figures 5A,B; “a”). Infected cells with a disrupted EIHM and an intact vacuole were typically viable (Figures 5A,B; “b”), however, some were dead based on nuclear PI labeling (Figures 5A,B; “c”). Conversely, infected cells with a disrupted EIHM and a ruptured vacuole were rarely viable (Figures 5A,B; “d”) with the majority appearing dead (Figures 5A,B; “e”). These results indicated that before IH spread into adjacent rice cells, the first-invaded rice cell died nearly concurrent with vacuole rupture. The association between vacuole rupture and cell death was further confirmed by time-lapse imaging (Figures 5C,D). Uninvaded rice cells that were adjacent to a dead first-invaded cell remained viable (Figure 5A; “e”).

### IH Undergo Transient Necrotrophic-Like Growth in the Dead First-Invaded Host Cell

**FIGURE 6:**
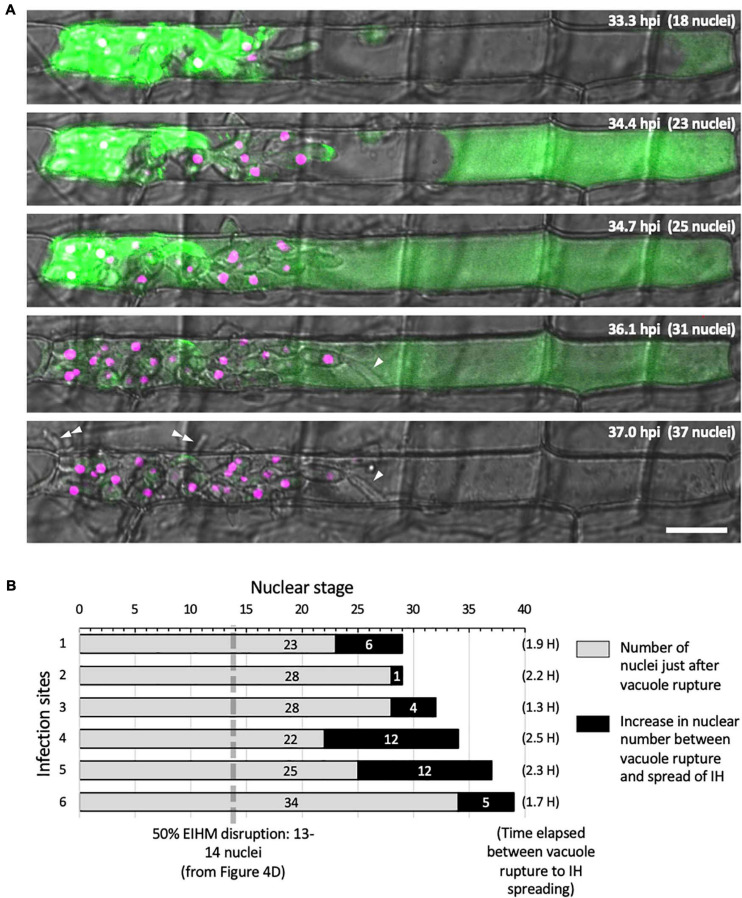
IH morphology changes after host cell death. **(A)** Representative time-lapse of *M. oryzae* CKF2187 expressing sec-GFP (green) and nuclear tdTomato (magenta) invading a rice cell from 33 to 37 hpi. Shown are single plane confocal images of merged bright-field and fluorescence. Nuclear stage is indicated in the upper right-hand corner together with hpi. Growth of IH become more filamentous after disruption of the vacuole (white arrowheads). The first IH to cross into the next host cell (double white arrowheads) originated from IH that had grown to be densely packed against the host cell wall before vacuole rupture. Bar = 20 μm. **(B)** Graphical summary showing six time-lapsed CKF2187 infections ranging from 32 to 40 hpi. Shown are the nuclear stages when vacuole rupture was observed (gray bars) and the relative increase in nuclear stage when IH were observed to spread into neighboring host cells (black bars). The time elapsed between vacuole rupture and IH spreading is shown in parenthesis, corresponding to the black bars. For additional context, the nuclear stage at which 50% EIHM disruption occurred (13–14 nuclei; Figure 4D; *n* = 390) is denoted by the dotted gray line.

Using sec-GFP and H1:tdTomato reporters to correlate the timing of vacuole rupture and IH growth stage in the first-invaded cell, we found that the vacuole ruptured at an average nuclear stage of 28 (Figure 6B; gray bars, range 22–34). The average then increased by six (Figure 6B; black bars; range 1–12) to an average nuclear stage of 34 when IH began to invade adjacent rice cells. The time that elapsed between rupture of the vacuole and the spread of IH into adjacent cells was 1.3–2.5 h (Figure 6B).

Intriguingly, the growth of IH within the dead host cell appeared morphologically distinct from bulbous IH; transitioning to growth that was more filamentous than the typical bulbous IH from which it arose (Figure 6A; white arrowheads). This transition was consistently observed throughout this study (Figures 2C, 5C). In addition, we noticed that the first IH to enter an adjacent host cell were from IH which had been closely associated with the rice cell wall before the vacuole ruptured (Figure 6A; white double arrowheads). Despite the proximity of these IH to the cell wall crossing point, they did not invade adjacent cells for over an hour after rupture of the vacuole. This suggested IH do not cross the rice cell wall into adjacent cells before the first-invaded cell dies.

### Dynamics of Effector Localization During Host Cell Invasion

**FIGURE 7:**
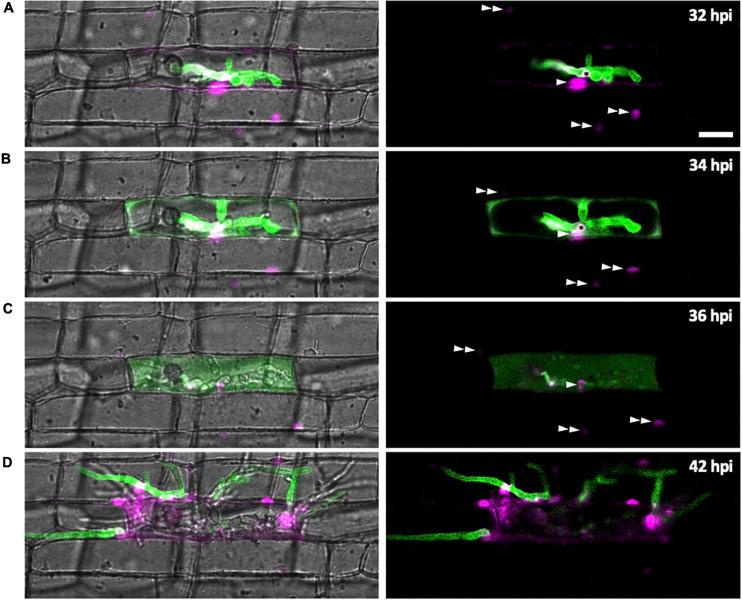
Effector localization changes during invasion of the first few host cells. *M. oryzae* CKF1616 expressing apoplastic effector Bas4:EGFP (green) along with cytoplasmic effector Pwl2:mCherry:NLS (magenta) invading rice. Shown are single plane merged fluorescence and bright-field (left panels), and merged fluorescence alone (right panels) confocal images of a time-lapse series from 32 to 42 hpi. Asterisk = BIC. Single white arrowhead = first-invaded host cell nucleus with Pwl2:mCherry:NLS fluorescence. Double white arrowhead = nuclei of uninvaded host cells with Pwl2:mCherry:NLS fluorescence. **(A)** During the early stage of invasion, the EIHM was still intact, causing Bas4:EGFP to be retained within the EIHMx. Pwl2:mCherry:NLS was localized at the BIC, in the nucleus of the invaded cell, and in the nuclei of a few nearby cells. **(B)** The EIHM was disrupted, causing Bas4:EGFP to spill into the rice cytoplasm. **(C)** The vacuole ruptured, causing spilled Bas4:EGFP to homogenize throughout the host cell lumen. **(D)** IH invaded neighboring cells with Bas4:EGFP retained by new EIHMs. Pwl2:mCherry:NLS fluorescence increased upon invasion of neighboring host cells. By this time the first-invaded cell lacks significant levels of fluorescence. Bar = 20 μm.

We investigated changes of subcellular localization of *M. oryzae* effectors during IH growth in first- and second-invaded cells using time-lapse imaging of rice cells invaded with *M. oryzae* strain CKF1616. This strain expresses apoplastic effector Bas4 fused to EGFP (Bas4:EGFP) together with cytoplasmic effector Pwl2 fused to mCherry and a nuclear localization signal (Pwl2:mCherry:NLS) (Khang et al., 2010). We reasoned that the localization patterns of these fusion proteins would also provide details regarding the state of PD permeability during *M. oryzae* invasion. The localization of these fusion proteins was consistent with a previous report (Khang et al., 2010). Specifically, in early invasion, Bas4:EGFP localized exclusively within the EIHMx around IH, indicating an intact EIHM. However, Pwl2:mCherry:NLS accumulated in three distinct subcellular structures: (1) the BIC, (2) the nucleus of the first-invaded rice cell, and (3) the nuclei of uninvaded neighboring rice cells (Figure 7A). At this early stage, the localization of Pwl2:mCherry:NLS indicated that Pwl2 is a cytoplasmic effector that is translocated into the rice cell cytoplasm, presumably through the BIC. Because this fusion protein contains an NLS signal, it is subsequently trafficked to the nuclei of rice cells. The accumulation of Pwl2:mCherry:NLS in the nuclei of neighboring cells suggested that PD were open at this early stage of rice cell invasion.

As IH continued to grow, the localization of Bas4:EGFP changed (Figure 7). During late invasion of the first rice cell, Bas4:EGFP moved from the EIHMx into the rice cytoplasm, indicating loss of EIHM integrity. Yet at this stage Bas4:EGFP did not appear to move into the adjacent rice cells. A consistent localization pattern was observed with sec-GFP (Figures 2, 4C, 5A,C and Supplementary Figures 1B, 2–4). The exclusion of Bas4:EGFP and sec:GFP from neighboring rice cells suggested closure of PD at later stages of invasion. Additionally, the BIC-localization of Pwl2:mCherry:NLS and EIHMx-localization of the Bas4:EGFP appeared in second-invaded cells (Figure 7D), a typical pattern observed in compatible interactions (Mosquera et al., 2009; Khang et al., 2010).

## Discussion

Plant-derived interfacial membranes are essential for the establishment and maintenance of biotrophy (Perfect and Green, 2001; Bozkurt et al., 2015), but little is known about the timing of interfacial membrane disruption, or what the consequences of this disruption are for both the pathogen and the infected host cell during hemibiotrophic invasion. In this study, we provided evidence that the EIHM in first-invaded cells is disrupted in a manner dependent on IH growth stage and that EIHM disruption is an integral part of a successful infection. We discuss additional features of successful *M. oryzae* infection in the following sections.

### Three Distinct Infection Phases During Early *Magnaporthe oryzae* Invasion

**FIGURE 8:**
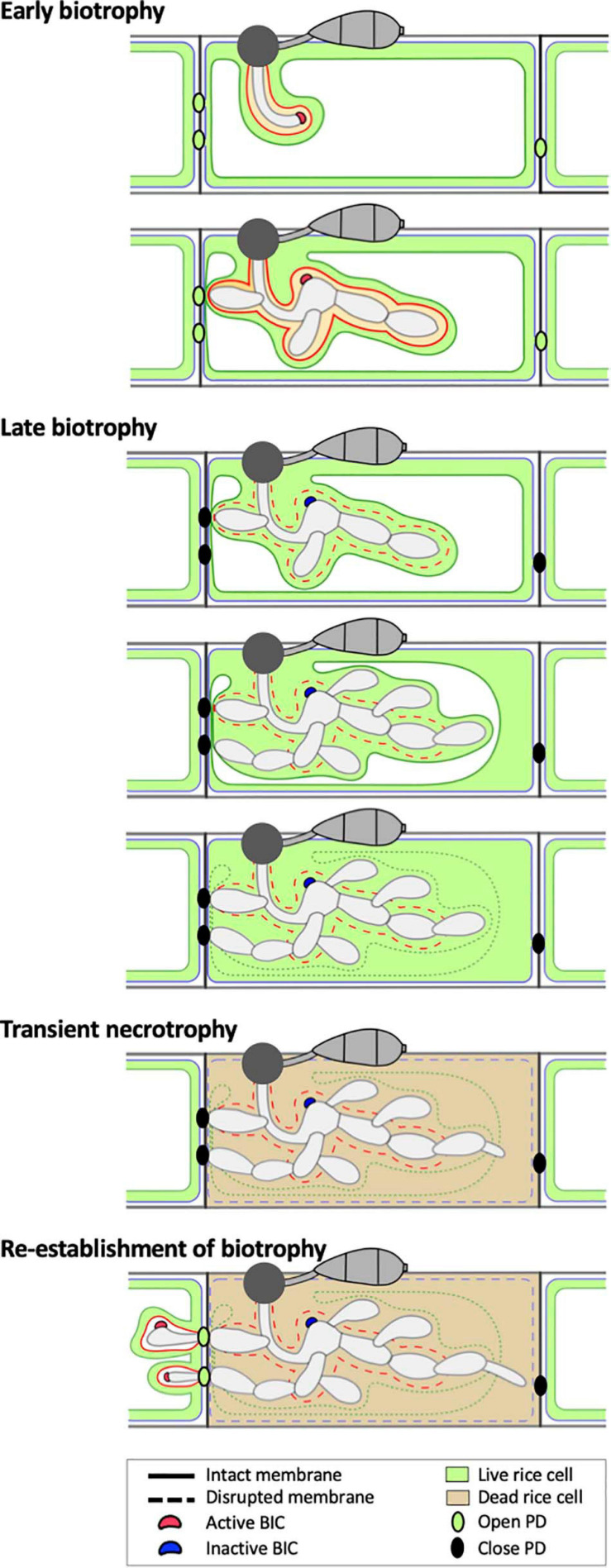
Model of the hemibiotrophic lifestyle of *M. oryzae* during invasion of the first and second rice cells. Early Biotrophy: An initial invasion of a rice cell is achieved by a filamentous primary hypha, which differentiates into the first bulbous IH cell. The tip BIC positioned at the apex of the primary hypha is left at a subapical position when the first bulbous cell differentiates. Branched bulbous IH then arise from both the first bulbous cell and from the primary hypha. All IH are encased by an intact EIHM. The EIHM-encased IH also invaginate the vacuole, resulting in a thin layer of cytoplasm on the rice-facing side of the EIHM. Apoplastic effectors are retained within the EIHMx, while cytoplasmic effectors accumulate at the BIC, enter the host cytoplasm, and move symplastically through open PDs into adjacent cells. Late biotrophy: The EIHM disrupts, causing apoplastic effectors to spill from the EIHMx into the host cytoplasm and exposing IH to direct contact with the host cell cytoplasm. By this time, effector cell-to-cell movement has ceased due to closed PDs. The host vacuole progressively shrinks around growing IH, resulting in increased cytoplasmic volume. This eventually ends in rupture of the vacuole, causing the cytoplasm and vacuolar contents to homogenously mix. Transient necrotrophy: The PM becomes permeabilized when the vacuole ruptures, resulting in host cell death. This occurs in a contained manner without affecting the viability of adjacent host cells. Leading IH then differentiate more filamentous growth, which lasts at least over an hour before invasion of adjacent host cells. Re-establishment of biotrophy: The first IH to invade a neighboring cell often originates from IH which have grown to be in close association with the rice cell wall before vacuole rupture. Invasion of adjacent cells is biotrophic with formation of new BICs and EIHM as well as invagination of the vacuole. Cytoplasmic and apoplastic effectors are again delivered to the cytoplasm and EIHMx, respectively.

We suggest that EIHM disruption and subsequent host cell death are landmarks that demarcate three distinct infection phases of initial *M. oryzae* invasion within the first-invaded rice cell: early biotrophy, late biotrophy, and transient necrotrophy (Figure 8). First, the early biotrophic phase maintains hallmarks typical of biotrophy, in which IH grow in living host cells while being surrounded by the intact EIHM. Second, the late biotrophic phase begins when the EIHM is disrupted, causing IH to grow with increasingly direct contact with the living host cytoplasm. During this phase the rice vacuole progressively shrinks and eventually disrupts, which coincides with death of the invaded cell marking the end of biotrophy. Third, the transient necrotrophic phase takes place within the dead host cell, ending when biotrophy is reestablished upon invasion of adjacent rice cells. During this necrotrophic phase, IH switch from bulbous to filamentous-like growth. The necrotrophy-like growth was transient. That is, IH grew within the dead host cell for ∼1.3 to 2.5 h (Figure 6), which is relatively brief compared with the ∼12 h IH spend colonizing the first-invaded cell (Kankanala et al., 2007). It is intriguing to note that IH were often closely associated with host cell walls during early/late biotrophic phases, but they did not move into adjacent cells for a minimum of 1 h after vacuolar rupture and host cell death (transient necrotrophic phase) (Figures 2, 5, 6). This suggests that the transient necrotrophic phase is required for IH cell-to-cell movement. Taken together, we propose that *M. oryzae* undergoes three distinct infection phases in each newly invaded cell during symptomless early invasion, and this lifestyle is followed by a complete transition to necrotrophy associated with macroscopic lesion development that typically occurs a few days after inoculation.

### EIHM Biogenesis and Subsequent Disruption

Considering the link between biotrophy and formation of the EIHM, understanding mechanisms of EIHM biogenesis is important. Kankanala et al. (2007) proposed that the EIHM is built *de novo* by redeploying host membranes toward the nascent rice-*M. oryzae* interface based on their FM4-64 dye-loading studies. These studies showed the dynamic association of host membrane tubules and round vesicles near the expanding EIHM. Recent studies with transgenic rice expressing GFP fusions with plasma membrane-localized proteins, such as OsCERK1, EL5, and LTi6b, demonstrated that the EIHM is continuous with the host PM but appears to be distinct from it. OsCERK1:GFP and EL5:GFP were typically present in the invaginated host plasma membrane surrounding young IH, but they were absent from the EIHM surrounding the mature bulbous IH. Conversely, GFP:LTi6b continuously outlined young and mature IH (Mentlak et al., 2012; Kouzai et al., 2014; Mochizuki et al., 2015). These results suggest that EIHM biogenesis begins with invagination of the host plasma membrane to surround early IH growth and subsequently transitions to *de novo* construction when IH differentiate bulbous growth. EIHM *de novo* construction likely involves the modulation of host membrane dynamics similar to those observed during interface biogenesis in other host-pathogen interactions (Koh et al., 2005; Micali et al., 2011; Bozkurt et al., 2015; Deeks and Sánchez-Rodríguez, 2016; Inada et al., 2016). The membrane source(s) and trafficking mechanism to build the EIHM remain unknown in the rice-*M. oryzae* interaction.

Like mechanisms of EIHM biogenesis, little is known about mechanisms disrupting EIHM integrity. In our study, we utilized nuclear number as a marker for IH development and as a proxy for the physical space occupied by the fungus within the first-invaded rice cell. We hypothesize that IH physically disrupt the EIHM as the fungus occupies increased space within the rice cell, and EIHM biogenesis is not able to rapidly assemble additional membrane. This hypothesis is supported by our results indicating that the EIHM starts to lose integrity at the tips of late-stage IH and that this integrity loss is irreparable (Figure 3). Alternatively, IH may secrete lytic enzymes at these late IH developmental stages that play a role in degrading the EIHM. For instance, *M. oryzae* may use a strategy similar to intracellular bacterial pathogen *Listeria monocytogenes* and protozoan pathogen *Toxoplasma gondii* that initially reside within a vacuole but later produce pore-forming proteins, Listeriolysin O and perforin-like protein 1, respectively, to disrupt the vacuole and reach the host cytoplasm (Kafsack et al., 2009; Hamon et al., 2012). It remains to be determined whether late-stage IH tips secrete pore-forming proteins to disrupt the EIHM and if, upon reaching the host cytoplasm, they contribute to permeabilization of the host vacuole membrane leading to gradual shrinkage and rupture of the vacuole. It may also be possible that *M. oryzae* and rice cells engage in a type of quorum-sensing like mechanism to detect the extent of IH development before initiating host cell death. However, additional research is needed to explore this possibility.

### Rice Cell Death During *Magnaporthe oryzae* Invasion

Our results showed that the viability loss of first-invaded rice cells coincided with the rupture of the central vacuole (Figure 5). Because the vacuole contains various hydrolytic enzymes, the rupture of the vacuole releases these enzymes into the cytoplasm where they degrade cellular organelles, eventually culminating in plant cell death (Jones, 2001; Hara-Nishimura and Hatsugai, 2011). The vacuole rupture is known to contribute to either disease resistance or susceptibility depending on pathogen lifestyle and the timing of the rupture relative to infection stage (Hatsugai et al., 2004; Hara-Nishimura and Hatsugai, 2011; Dickman and Fluhr, 2013; Mochizuki et al., 2015). Mochizuki et al. (2015) used transgenic rice expressing vacuole membrane-localized GFP and showed that the vacuole gradually shrank and eventually ruptured in susceptible *M. oryzae*-invaded rice cells, consistent with our results. They further demonstrated that vacuole rupture caused critical damage to non-branched IH at the early infection stage but not to branched IH at the later infection stage, suggesting a fungal-driven mechanism for maintaining the integrity of the host vacuole until IH gain tolerance to vacuole disruption. Vacuole-mediated cell death in plants is regulated by vacuolar processing enzymes (VPEs) (Hatsugai et al., 2006, 2015). In rice, five VPE (OsVPE) genes have been identified, and the expression levels of OsVPE2 and OsVPE3 were shown to increase during reactive oxygen species (ROS)-induced vacuole rupture and cell death (Deng et al., 2011; Christoff et al., 2014). We propose that death of first-invaded rice cells during susceptible, or compatible, *M. oryzae*-rice interactions is vacuole-mediated, likely involving OsVPEs.

Recently, ferroptotic cell death was identified during resistant, or incompatible, *M. oryza*e-rice interactions (Dangol et al., 2019). Ferroptotic cell death is a distinct form of cell death that typically prevents successful fungal invasion of neighboring rice cells (Yang and Stockwell, 2016; Dangol et al., 2019). A key event in ferroptotic cell death is the accumulation of ROS and iron (Fe^3+^) within the first-invaded rice cell during incompatible interactions (Dangol et al., 2019). Notably, in compatible interactions, ROS and iron failed to accumulate within the first-invaded rice cell at 30 and 48 hpi (Dangol et al., 2019). Although both compatible and incompatible interactions involve ROS production, compatible interactions do not lead to extensive ROS accumulation within the first-invaded rice cell, a key feature of ferroptosis (Liu et al., 2020). In our study, we did not monitor ROS accumulation within the rice cell during compatible fungal-rice interactions. It is likely that initial ROS production occurs during the early biotrophic phase when the EIHM is intact. Further research is needed to examine the timing and extent of ROS production within the context of the early biotrophy, late biotrophy, and subsequent transient necrotrophy. These details will aid in discerning processes of rice cell death during compatible and incompatible interactions.

### Infection Phase-Specific Dynamics of *M. oryzae* Effector Proteins

This study provides evidence that subcellular localization of *M. oryzae* effectors change during IH growth in first-invaded cells (Figure 7). During the early biotrophic phase, fluorescently-tagged effectors Bas4 (apoplastic effector) and Pwl2 (cytoplasmic effector) exhibited distinct localization patterns; Bas4 accumulated in EIHMx, whereas Pwl2 preferentially accumulated in BICs, entered the host cytoplasm, and moved into surrounding cells (Figure 7), consistent with previous results (Khang et al., 2010). Bas4 was then dramatically relocalized during the late biotrophic phase when the EIHM was disrupted; spilling from the EIHMx into the host cytoplasm. These results suggested that all *M. oryzae* effectors eventually enter the host cytoplasm either by translocation across the intact EIHM (cytoplasmic effectors) during the early biotrophic phase or by spilling through the disrupted EIHM (apoplastic effectors) during the late biotrophic phase. Apoplastic effector re-localization has significant implications in the study of effectors: (1) Effectors are generally classified into either cytoplasmic effectors or apoplastic effectors, depending on their localization in the host cytoplasm or in the apoplast (interfacial compartment or intercellular space), respectively (Schornack et al., 2009; Giraldo and Valent, 2013; Lo Presti and Kahmann, 2017). We suggest that these effector classifications, however, must be defined in the context of infection stages, at least for *M. oryzae* effectors such as Bas4 that are localized in the EIHM compartment but are subsequently re-localized in the host cytoplasm; (2) Live-cell imaging of *M. oryzae* expressing fluorescently-tagged effectors has been instrumental for determining the identity of an effector as apoplastic or cytoplasmic and for investigating the mechanism by which cytoplasmic effectors are translocated into the host cytoplasm (Mosquera et al., 2009; Khang et al., 2010; Mentlak et al., 2012; Park et al., 2012; Ribot et al., 2013; Nishimura et al., 2016; Sharpee et al., 2017). This approach requires that individual infection sites be assessed for EIHM integrity. The use of sec-GFP would be one example approach to differentiate effector translocation across the intact EIHM from effector spillage through the disrupted EIHM, as suggested by earlier studies (Mosquera et al., 2009; Khang et al., 2010; Giraldo et al., 2013). Given the intimate link between effector localization and function (Giraldo and Valent, 2013; Sharpee et al., 2017), *M. oryzae* apoplastic effectors may play roles in both the apoplast and the cytoplasm, depending on their stage-specific localization. Three *M. oryzae* apoplastic effectors, Slp1, Bas4, and Bas113, have been shown to localize in the EIHMx, and Slp1 was further determined to function as a LysM protein that sequesters chitin to suppress host immunity (Mosquera et al., 2009; Mentlak et al., 2012; Giraldo et al., 2013). It is an exciting possibility that these and many yet-to-be-identified apoplastic effectors may have host targets in both the apoplast and the cytoplasm.

Although the mechanism of how effectors are translocated across the interfacial membrane remains unknown for most filamentous pathogens, increasing evidence suggests that BICs formed in *M. oryzae*-invaded rice cells function as the site of translocation into the rice cytoplasm across the intact EIHM (Khang et al., 2010; Giraldo et al., 2013). We propose that BICs undergo three developmental and functional stages in first-invaded cells: in the first stage, a single “tip-BIC” appears at the tip of the filamentous primary IH; in the second stage, the tip BIC becomes the “early side-BIC” when the filamentous hypha differentiates bulbous growth; in the third stage, the early side-BIC remains on the side of the first bulbous IH cell as the “late side-BIC” while IH continue to proliferate in the rice cell (Figure 8). Host cytoplasmic dynamics appear to be focused in the vicinity of the tip BIC and the early side-BIC. The tip and early-side BICs strongly accumulate fluorescently-tagged cytoplasmic effectors, whereas the late side-BIC shows weaker intensity of effector-associated fluorescence (Khang et al., 2010; Jones et al., 2016b; Kim and Khang, unpublished data). We hypothesize that the tip- and the early side-BICs are actively performing their presumed function in effector delivery during the early biotrophic phase when the EIHM is intact, and the late side-BIC is a remnant that has ceased to deliver effectors. This is consistent with evidence that expression of the BIC-localized cytoplasmic effector gene *PWL2* is strongly induced at early infection stages when the tip- and early side-BIC are present (Zhu et al., 2021). A future research tool that can directly demonstrate the role of BICs in effector translocation is needed to test this hypothesis. It has been an intriguing question why there is only a single BIC in each first-invaded cell. It may be because the BIC is required for the delivery of cytoplasmic effectors into host cells only during the early biotrophic phase while the EIHM is intact; the BIC becomes obsolete once the EIHM is disrupted.

### Infection Phase-Specific Changes in the Plasmodesmata Permeability

Previous studies suggest that *M. oryzae* exploits open PD for cell-to-cell movement of effectors and of IH during biotrophic invasion (Kankanala et al., 2007; Khang et al., 2010; Sakulkoo et al., 2018). Our results expand these studies and further suggest that PD permeability changes in a manner specific to infection phase. At the early biotrophic invasion stage, Pwl2:mCherry:NLS (44.5 kD) entered the host cytoplasm across the intact EIHM and localized within the nucleus of the first-invaded cell, as well as, nuclei of adjacent cells (Figure 7A). This successful movement into neighboring cells suggests PD are open. However, during later stages, Bas4:EGFP (36 kD) and sec-GFP (26.9 kD) accumulated within the first-invaded cell following loss of EIHM integrity, indicating PD are closed (Figures 7B,C). If PD were open at these later invasion stages, we would expect the successful movement of Bas4:EGFP and sec-GFP through PD into adjacent cells because the larger fusion protein, Pwl2:mCherry:NLS, moved into the nuclei of uninvaded rice cells at the early invasion stage. We propose that infection phase-dependent PD dynamics are integral to *M. oryzae*’s successive biotrophic invasion. That is, during the early biotrophic phase, PD serve as a conduit for cytoplasmic effectors to move into surrounding uninvaded rice cells where they are presumed to prepare rice cells for invasion (Khang et al., 2010). During the subsequent infection phases when the viability of the first-invaded cell declines and is eventually lost, PD become closed, which prevents death signals from spreading into uninvaded cells, thus keeping these cells unaffected and viable. Subsequently, PD are exploited by IH to move into adjacent viable host cells (Kankanala et al., 2007; Sakulkoo et al., 2018).

How PD permeability is regulated during rice blast disease remains an open question. It is generally known that pathogen infections induce PD closure by recruiting PD-associated molecules such as callose (a β-1,3 glucan polymer) and that such PD closure is linked to host immunity (Lee, 2015). During rice colonization by *M. oryzae*, callose deposition at PD is dynamic and linked to infection progression (Sakulkoo et al., 2018). In the first-invaded cell, callose papillae are typically associated with sites of appressorium-mediated penetration but do not appear at PD until later stages of rice infection, consistent with onset of cell death in the first-invaded rice cell (Sakulkoo et al., 2018). Studies in *Arabidopsis thaliana* suggest that recognition of chitin (pathogen-associated molecular pattern, PAMP, from fungal pathogens) by the chitin pattern recognition receptor LYM2 (also known as AtCEBiP) leads to PD closure (Faulkner et al., 2013) and also that the PD-localized protein 5 (PDLP5) mediates callose deposition at PD in a manner depending on the defense hormone salicylic acid (Lee and Lu, 2011). It is an interesting possibility that PAMP-triggered PD closure in rice is suppressed when IH grow within the EIHM (the early biotrophic phase) but is then activated when IH are exposed to the host cytoplasm after the EIHM disrupts, which might result in increased PAMP recognition by rice PRRs (the late biotrophic phase). Mentlak et al. (2012) showed that *M. oryzae* secretes the apoplastic effector Slp1 to sequester chitin released from IH growing within the EIHM and thus prevents chitin from being recognized by the rice chitin PRR CEBiP. It remains to be determined whether *M. oryzae* Slp1 and rice PRRs, including CEBiP, play a role in PD regulation during rice blast disease. Although the precise mechanism of how IH cross the cell wall to invade adjacent cells after the transient necrotrophic phase remains unknown, it may involve modulation of closed PD, for example degrading PD callose using hydrolytic enzymes such as β-1, 3–glucanases and β-glucosidases to reopen them. Understanding how PD permeability is regulated during *M. oryzae* invasion and how the PD dynamics is linked to host susceptibility and resistance will offer potential targets that can be exploited to control blast disease.

## Materials and Methods

### Strains, Fungal Transformation, and Plasmid Construction

*Magnaporthe oryzae* wild-type strain O-137, isolated from rice (*Oryza sativa*) in China (Orbach et al., 2000), was used as a recipient strain to generate fungal transformants using *Agrobacterium tumefaciens*-mediated transformation (Khang et al., 2006). We used the rice strain YT-16 highly susceptible to *M. oryzae* O-137 (Kankanala et al., 2007) and all O-137-derived transformants used in this study. See Supplementary Table 1 for the list of *M. oryzae* transformants. The Dendra2 gene was PCR-amplified from tol2-mpx-Dendra2 [a gift from Anna Huttenlocher; Addgene plasmid # 29574; (Yoo and Huttenlocher, 2011)] using the primers CKP303: 5′-GGATCCATGAACACCCCGGGAATTAAC-3′ and CKP304: 5′-TGTACAGCCACACCTGGCTGGG-3′, underlined for *Bam*HI and *Bsr*GI sites, respectively. The *BAS4* promoter and its entire 102-amino acid coding sequence [1.3 kb *Eco*RI-*Bam*HI fragment (Khang et al., 2010)] and Dendra2 were cloned together with the Nos terminator [0.3 kb *Bsr*GI-*Sal*I fragment from pBV360 (same as pAN583; Nelson et al., 2007)] in the binary vector pBGt to generate pCK1244 (*BAS4*:Dendra2:Terminator). The *BAS4* promoter and signal peptide-encoding sequence were cloned together with the *EGFP* and the *Neurospora crassa* β-tubulin gene terminator in the binary vector pBHt2 to generate pBV324 (sec-GFP construct) (Khang et al., 2010). The *M. oryzae* ribosomal protein 27 gene (P27) promoter was used to construct the constitutive expression plasmid pCK1292 for cytoplasmic tdTomato (Jones et al., 2016b). The EGFP gene was obtained from Clontech, and the tdTomato was isolated from pAN582 (Nelson et al., 2007). The P27 promoter and histone H1 gene from *N. crassa*, which was isolated from pBV229 (Shipman et al., 2017), was cloned together with tdTomato at the upstream of the sec-GFP construct in pBV324 to generate pCK1312. See Supplementary Table 2 for the list of plasmids used.

### Infection Assays

Rice sheath inoculations were performed as previously described (Kankanala et al., 2007). Briefly, excised leaf sheaths (5–9 cm long) from 2 to 3 weeks old plants were inoculated by injecting a spore suspension (5 × 10^4^ spores/ml in sterile water) into the hollow interior of the sheath. The inner epidermal layer of the inoculated sheath was hand-trimmed for confocal microscopy.

### Staining and Plasmolysis

Propidium iodide (PI) was prepared to a 10 μg/ml working solution by diluting 10 μl of stock solution (catalog No. P3566; 10 ml of 1 mg/ml solution in water; ThermoFisher) in 990 μl of water. Trimmed leaf sheaths were submerged in the PI working solution for 15 min and then mounted in the same solution for microscopy. FM4-64 was prepared to a 17 mM aqueous stock solution by adding 9.2 μl of sterile distilled water to 100 μg of FM4-64 powder (catalog No. T13320; 10 × 100 μg; ThermoFisher) and stored at −20°C. Trimmed leaf sheaths were incubated in a 17 mM aqueous working solution for 1 h, washed with water, and then incubated for four more hours prior to microscopy. Fluorescein diacetate (FDA; catalog No. F7378, 5 g powder; Sigma) was dissolved in acetone to make a stock concentration of 1 mg/ml. A working solution of FDA (2 μg/ml, 0.2% acetone) was prepared by diluting 2 μl of the stock solution in 1 ml of water. Sucrose-induced plasmolysis was performed by replacing the mounting solution of water with a 0.5 M sucrose solution and incubated for 25 min before microscopy.

### Confocal Microscopy

Confocal microscopy was performed on a Zeiss Axio Imager Z1 inverted microscope equipped with a Zeiss LSM 710 system using Plan-Apochromat 20 × /0.8 NA and Plan-Neofluor 40 × /1.3 NA (oil) objectives. Excitation/emission wavelengths were 488 nm/496 to 544 nm for GFP and fluorescein, 543 nm/565 to 617 nm for mCherry and tdTomato, 543 nm/580 to 640 nm for PI, and 543 nm/613 to 758 nm for FM4-64. Images were acquired using the Zen Black 2011 software. Images were processed using the Zen Black software (version 10.0, Zeiss). For long interval time-lapse imaging, the coverslip was removed and water was added to the slide in between images to prevent dehydration and to allow gas exchange to occur. Selective photoconversion of Bas4:Dendra2 was performed by irradiating a region of interest with the 405 nm laser line (100% output power and a pixel dwell time of 1.58 μs with 250 iterations) using the 40x objective lens at a zoom factor of 2. Excitation/emission wavelengths for imaging unconverted green Dendra2 were 488 nm/496 to 554 nm and 543 nm/560 to 675 nm for imaging converted red Dendra2.

## Data Availability Statement

The original contributions presented in the study are included in the article/Supplementary Material, further inquiries can be directed to the corresponding author.

## Author Contributions

CK conceived and designed the experiments. KJ, JZ, CJ, and DK performed the experiments. KJ, JZ, CJ, DK, MP, and CK analyzed the data and wrote the manuscript. All authors contributed to the article and approved the submitted version.

## Conflict of Interest

The authors declare that the research was conducted in the absence of any commercial or financial relationships that could be construed as a potential conflict of interest.

## Supplementary Material

Supplementary Figure 1 FM4-64 labels IH septa only when sec-GFP is spilled into the host cell. **(A,B)** *M. oryzae* CKF2180 (sec-GFP; green) invading rice cells. Inoculated sheaths were pulse-stained with FM4-64 (shown in magenta) for 1 h, washed with water, and then incubated for 4 h prior to microscopy. Shown are single plane merged fluorescence, split fluorescence, and bright-field confocal images. Bars = 20 μm (full size images) and 5 μm (insets). **(A)** An infection at 28 hpi shows sec-GFP exclusively outlining IH. Inset shows a region of IH enlarged to demonstrate the absence of FM4-64 labeling (pseudo-colored white) near the septum (white arrow). Both EIHMx-localized sec-GFP and the absence of FM4-64 labeling from IH septa were consistent with an intact EIHM preventing the diffusion of either fluorophore. **(B)** A different infection at 32 hpi shows sec-GFP spilled into the host cell. Inset shows a region of IH enlarged to show positive FM4-64 labeling of fungal membranes at three septa (white arrows). Both the host-localized sec-GFP and fungal labeling of FM4-64 were consistent with a disrupted EIHM.

Supplementary Figure 2 Sec-GFP spills into the rice cytoplasm after EIHM disruption. **(A,B)** *M. oryzae* CKF1996 expressing sec-GFP (green) and cytoplasmic tdTomato (magenta) invading rice. Shown are single plane confocal images of both merged fluorescence and bright-field [**(A)**, top; **(B)**, left], or bright-field alone [**(A)**, bottom], and a merged fluorescence projection of 15 z-slices with 2 μm each [**(B)**, right]. Bars = 20 μm. **(A)** Infection at 30 hpi with sec-GFP spilled into the host cell, indicating the EIHM was disrupted. The vacuole membrane is visible in the bright-field (white arrows). **(B)** Infection at 32 hpi with sec-GFP in the host cell. After the top image was taken water was replaced with 0.5 M sucrose to induce plasmolysis. After 25 min, host-localized sec-GFP was retracted from the cell wall and remained excluded from the vacuole, demonstrating that sec-GFP was indeed localized within the host cytoplasm. Note that IH shifted slightly after the host cell was plasmolyzed. **(C)** *M. oryzae* CKF3267 expressing secreted mCherry (magenta) invading a rice cell between 31 and 33 hpi. Shown are single plane merged or split fluorescence confocal images of the same infection site. Like sec-GFP, secreted mCherry spilled into the host cytoplasm (top), indicating a disrupted EIHM. The rice sheath was then stained with 0.2 μg/ml FDA. FDA is converted to its fluorescent form in the rice cytoplasm where it is then retained (Jones et al., 2016b). FDA fluorescence (green) co-localized with secreted mCherry in the host cell, confirming cytoplasmic localization of spilled mCherry (middle). This was further confirmed by subsequently inducing plasmolysis with 0.5 M sucrose, which caused retraction of the colocalized spilled mCherry and FDA fluorescence from the cell wall as expected (bottom). The time elapsed between each image was 30 min. Bar = 20 μm.

Supplementary Figure 3 Image analysis – Brightness and contrast adjustment to reveal instances of low-intensity sec-GFP fluorescence in the host cell. During the initial stages of our quantitative analysis of sec-GFP localization and nuclear stage, we discovered that some infected host cells contained low-intensity green fluorescence in the cytoplasm that was impossible or nearly impossible to detect at the default display setting. Infections showing this pattern were prone to being misinterpreted as possessing an intact EIHM unless a more detailed image analysis was performed. To maximize the sensitivity of our EIHM integrity assay we empirically derived an appropriate adjustment to the image display settings in the Zen software (black edition) that consistently revealed instances of low intensity green fluorescence in the host cytoplasm (Figures 4C,D; *n* = 390). Ultimately we found that sufficient brightness and contrast for resolving background noise and low-intensity green fluorescence was achieved by adjusting the white point in the green fluorescence channel histogram from the default maximum of 4,095 (for 12-bit images) [**(A);** left] to 200 [**(A);** right]. This produced a display of the image where pixel intensity values 0–200 were proportionally increased in intensity in order to populate the full dynamic range (grayscale), while values 201–4,096 were displayed as saturated. Once the new white point was applied to an image, individual z-stacks were inspected for presence of host-localized green fluorescence at low-intensity. Shown in panel **(B)** through panel **(D)** are single plane merged bright-field and fluorescence confocal images of *M. oryzae* CKF2187 infections expressing sec-GFP (green) and H1:tdTomato (magenta) during invasion of the first rice cell between 29 and 31 hpi. Each shows a representative outcome of the image analysis. At default display settings, infection **(B)** appeared to have EIHMx-exclusive sec-GFP localization, indicating an intact EIHM. After the white point was lowered in the green channel, the sec-GFP localization pattern was confirmed as EIHMx-exclusive. Similar to panel **(B)**, infection **(C)** initially appeared to have an intact EIHM at the default display setting. However, after reducing the white point, low-intensity sec-GFP fluorescence was revealed to be present in the host cytoplasm, thus reversing the initial scoring and highlighting the importance of careful image analysis. Infection **(D)** was readily discernable as having a disrupted EIHM with both the default and adjusted display settings. Note that infections with this pattern (host-localized sec-GFP, disrupted vacuole) were usually identifiable with the default display. Bar = 20 μm, scale is equivalent for all images.

Supplementary Figure 4 Variation in host-localized sec-GFP fluorescence patterns. Our quantitative analysis of sec-GFP (green) localization in the context of nuclear stage (magenta) for *M. oryzae* CKF2187 infections between 28 and 33 hpi revealed 155 infections (out of 390) with host-localized sec-GFP (Figure 4D). The majority of these patterns were cytoplasmic (44.5%) or homogenous throughout the rice cell (48.4%) with the remaining 7.1% showing sec-GFP fluorescence: (1) inside only the vacuole (1.3%), (2) in both the cytoplasm and vacuole with higher intensity in the cytoplasm (3.2%), (3) in both the cytoplasm and vacuole with higher intensity in the vacuole (0.7%), and (4) ambiguous host-localization (1.9%). Together, these data indicated that spilled sec-GFP was typically found to be cytoplasmic or homogeneous, however, it could occasionally spill into the vacuole, or other combinations of host compartments. Shown are single plane merged fluorescence and bright-field confocal images of representative CKF2187 infections for each variation of host-localized sec-GFP fluorescence. Bars = 20 μm.

Supplementary Table 1 *Magnaporthe oryzae* transformants used in this study.

Supplementary Table 2 Key plasmids used in this study.
