# Supplementary figures and images for: Disruption of the Interfacial Membrane Leads to Magnaporthe oryzae Effector Re-location and Lifestyle Switch During Rice Blast Disease
Source: Front Cell Dev Biol. 2021 Jun 17;9:681734. doi: 10.3389/fcell.2021.681734 (PMC8248803; doi:10.3389/fcell.2021.681734)

Supplementary Figure 1

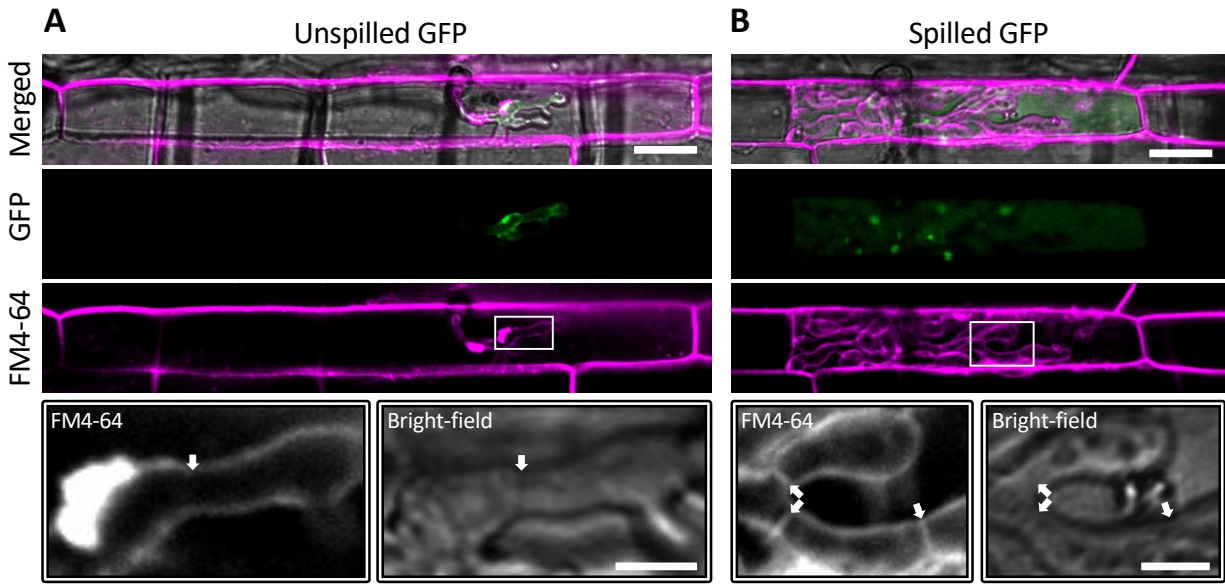

Supplement: Supplementary Figure 1 — FM4-64 labels IH septa only when sec-GFP is spilled into the host cell. (A,B) M. oryzae CKF2180 (sec-GFP; green) invading rice cells. Inoculated sheaths were pulse-stained with FM4-64 (shown in magenta) for 1 h, washed with water, and then incubated for 4 h prior to microscopy. Shown are single plane merged fluorescence, split fluorescence, and bright-field confocal images. Bars = 20 μm (full size images) and 5 μm (insets). (A) An infection at 28 hpi shows sec-GFP exclusively outlining IH. Inset shows a region of IH enlarged to demonstrate the absence of FM4-64 labeling (pseudo-colored white) near the septum (white arrow). Both EIHMx-localized sec-GFP and the absence of FM4-64 labeling from IH septa were consistent with an intact EIHM preventing the diffusion of either fluorophore. (B) A different infection at 32 hpi shows sec-GFP spilled into the host cell. Inset shows a region of IH enlarged to show positive FM4-64 labeling of fungal membranes at three septa (white arrows). Both the host-localized sec-GFP and fungal labeling of FM4-64 were consistent with a disrupted EIHM. [file Image_1.pdf]

Supplementary Figure 2

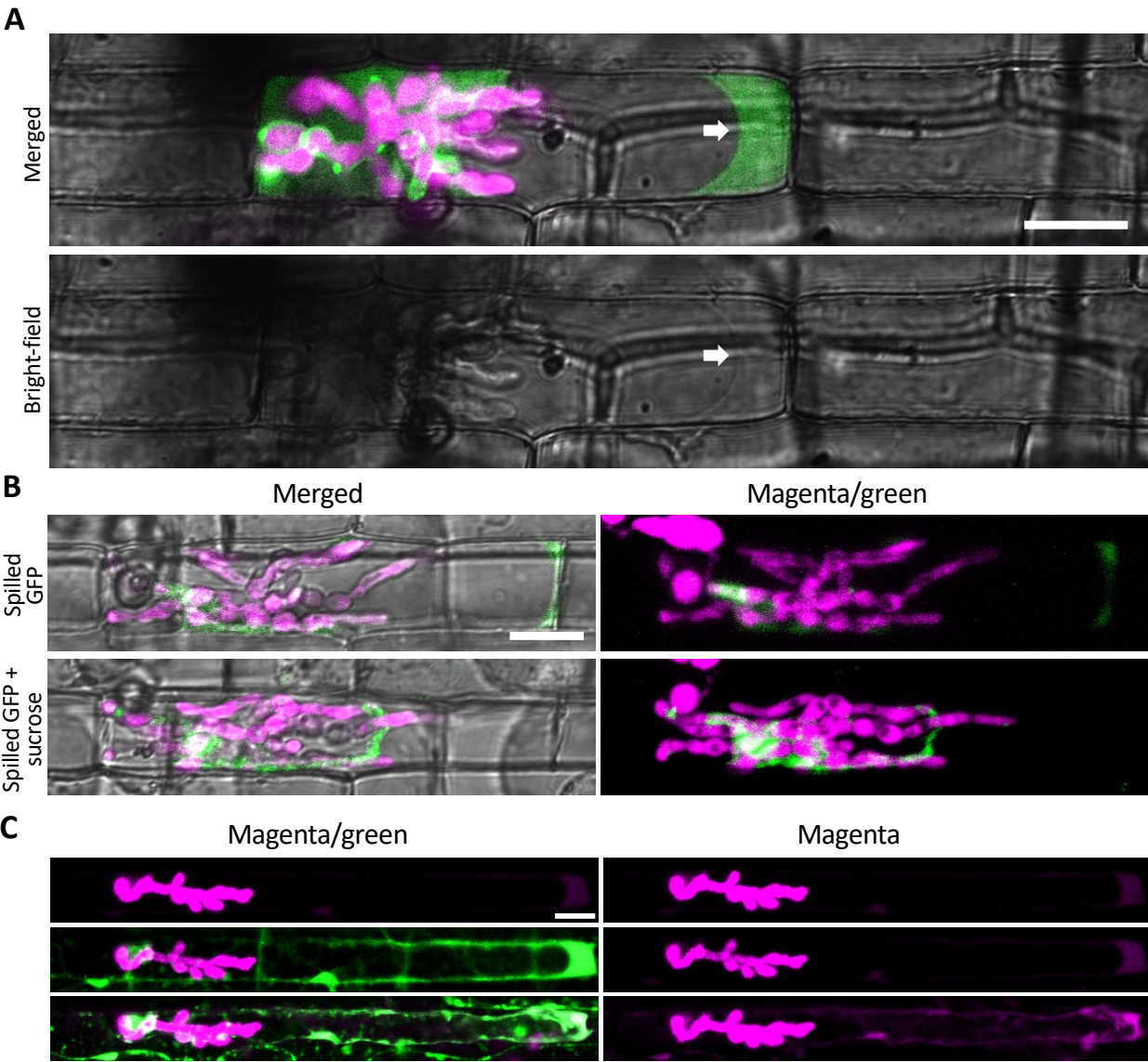

Supplement: Supplementary Figure 2 — Sec-GFP spills into the rice cytoplasm after EIHM disruption. (A,B) M. oryzae CKF1996 expressing sec-GFP (green) and cytoplasmic tdTomato (magenta) invading rice. Shown are single plane confocal images of both merged fluorescence and bright-field [(A), top; (B), left], or bright-field alone [(A), bottom], and a merged fluorescence projection of 15 z-slices with 2 μm each [(B), right]. Bars = 20 μm. (A) Infection at 30 hpi with sec-GFP spilled into the host cell, indicating the EIHM was disrupted. The vacuole membrane is visible in the bright-field (white arrows). (B) Infection at 32 hpi with sec-GFP in the host cell. After the top image was taken water was replaced with 0.5 M sucrose to induce plasmolysis. After 25 min, host-localized sec-GFP was retracted from the cell wall and remained excluded from the vacuole, demonstrating that sec-GFP was indeed localized within the host cytoplasm. Note that IH shifted slightly after the host cell was plasmolyzed. (C) M. oryzae CKF3267 expressing secreted mCherry (magenta) invading a rice cell between 31 and 33 hpi. Shown are single plane merged or split fluorescence confocal images of the same infection site. Like sec-GFP, secreted mCherry spilled into the host cytoplasm (top), indicating a disrupted EIHM. The rice sheath was then stained with 0.2 μg/ml FDA. FDA is converted to its fluorescent form in the rice cytoplasm where it is then retained (Jones et al., 2016b). FDA fluorescence (green) co-localized with secreted mCherry in the host cell, confirming cytoplasmic localization of spilled mCherry (middle). This was further confirmed by subsequently inducing plasmolysis with 0.5 M sucrose, which caused retraction of the colocalized spilled mCherry and FDA fluorescence from the cell wall as expected (bottom). The time elapsed between each image was 30 min. Bar = 20 μm. [file Image_2.pdf]

Supplementary Figure 3

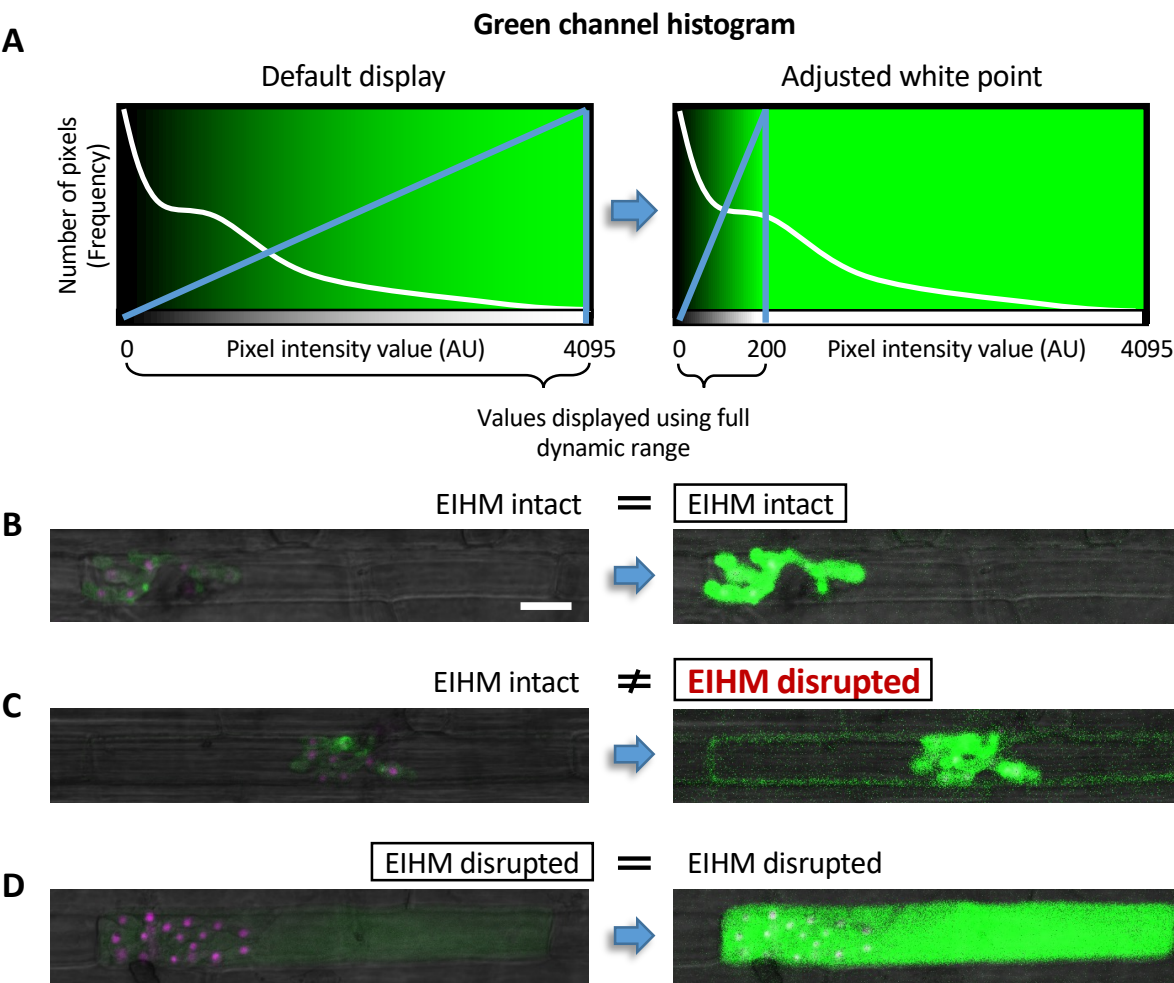

Supplement: Supplementary Figure 3 — Image analysis – Brightness and contrast adjustment to reveal instances of low-intensity sec-GFP fluorescence in the host cell. During the initial stages of our quantitative analysis of sec-GFP localization and nuclear stage, we discovered that some infected host cells contained low-intensity green fluorescence in the cytoplasm that was impossible or nearly impossible to detect at the default display setting. Infections showing this pattern were prone to being misinterpreted as possessing an intact EIHM unless a more detailed image analysis was performed. To maximize the sensitivity of our EIHM integrity assay we empirically derived an appropriate adjustment to the image display settings in the Zen software (black edition) that consistently revealed instances of low intensity green fluorescence in the host cytoplasm (Figures 4C,D; n = 390). Ultimately we found that sufficient brightness and contrast for resolving background noise and low-intensity green fluorescence was achieved by adjusting the white point in the green fluorescence channel histogram from the default maximum of 4,095 (for 12-bit images) [(A); left] to 200 [(A); right]. This produced a display of the image where pixel intensity values 0–200 were proportionally increased in intensity in order to populate the full dynamic range (grayscale), while values 201–4,096 were displayed as saturated. Once the new white point was applied to an image, individual z-stacks were inspected for presence of host-localized green fluorescence at low-intensity. Shown in panel (B) through panel (D) are single plane merged bright-field and fluorescence confocal images of M. oryzae CKF2187 infections expressing sec-GFP (green) and H1:tdTomato (magenta) during invasion of the first rice cell between 29 and 31 hpi. Each shows a representative outcome of the image analysis. At default display settings, infection (B) appeared to have EIHMx-exclusive sec-GFP localization, indicating an intact EIHM. After the white point was lower [file Image_3.pdf]
