# Supplementary material for: Disruption of the Interfacial Membrane Leads to Magnaporthe oryzae Effector Re-location and Lifestyle Switch During Rice Blast Disease
Source: Front Cell Dev Biol. 2021 Jun 17;9:681734. doi: 10.3389/fcell.2021.681734 (PMC8248803; doi:10.3389/fcell.2021.681734)
Supplement: Supplementary Figure 4 — Variation in host-localized sec-GFP fluorescence patterns. Our quantitative analysis of sec-GFP (green) localization in the context of nuclear stage (magenta) for M. oryzae CKF2187 infections between 28 and 33 hpi revealed 155 infections (out of 390) with host-localized sec-GFP (Figure 4D). The majority of these patterns were cytoplasmic (44.5%) or homogenous throughout the rice cell (48.4%) with the remaining 7.1% showing sec-GFP fluorescence: (1) inside only the vacuole (1.3%), (2) in both the cytoplasm and vacuole with higher intensity in the cytoplasm (3.2%), (3) in both the cytoplasm and vacuole with higher intensity in the vacuole (0.7%), and (4) ambiguous host-localization (1.9%). Together, these data indicated that spilled sec-GFP was typically found to be cytoplasmic or homogeneous, however, it could occasionally spill into the vacuole, or other combinations of host compartments. Shown are single plane merged fluorescence and bright-field confocal images of representative CKF2187 infections for each variation of host-localized sec-GFP fluorescence. Bars = 20 μm. [file Image_4.pdf]

Supplementary Figure 4

**Spill pattern**	**Representative infection**	**# out of 155 infections**	**% of infections**
Cytoplasmic	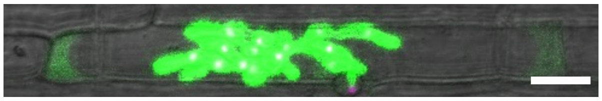	69	44.5
Homogenous	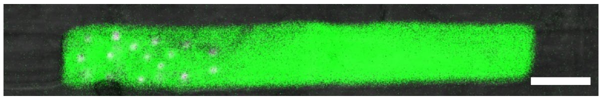	75	48.4
Vacuolar	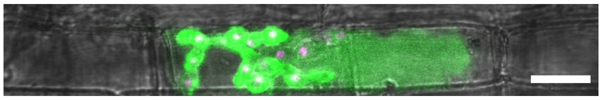	2	1.3
Cytoplasmic with some in vacuole	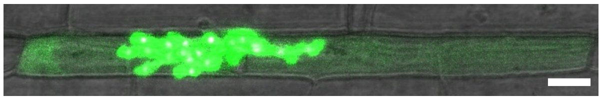	5	3.2
Vacuolar with some in cytoplasm	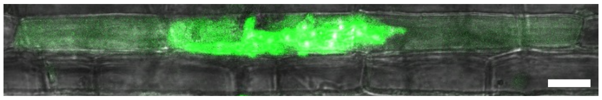	1	0.7
Ambiguous	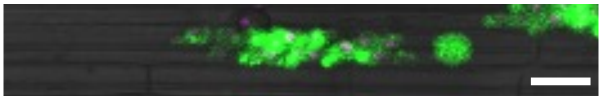	3	1.9
