# Supplementary material for: Disruption of the Interfacial Membrane Leads to Magnaporthe oryzae Effector Re-location and Lifestyle Switch During Rice Blast Disease
Source: Front Cell Dev Biol. 2021 Jun 17;9:681734. doi: 10.3389/fcell.2021.681734 (PMC8248803; doi:10.3389/fcell.2021.681734)
Supplement: Supplementary Table 1 — Magnaporthe oryzae transformants used in this study. [file Table_1.pdf]

Supplementary Table 1. ***Magnaporthe oryzae*** **transformants used in this study**

Strain	Used in	Description [Recipient strain; Plasmid used]
CKF315[a]	Figures 5A and 5C	Transformant expressing the Bas4 signal peptide fused to EGFP under control of the *BAS4* promoter [O-137[b]; pBV324]; same as KV97 (Mosquera et al., 2009)
CKF1616	Figure 7	Transformant expressing both a fusion of the Pwl2 coding sequence with mCherry:NLS under control of the *PWL2* promoter and a fusion of the Bas4 coding sequence with EGFP under control of the *BAS4* promoter [O-137; pBV591]; same as KV121 (Khang et al., 2010)
CKF1737	Figure 1B	Transformant expressing a fusion of the Bas4 coding sequence with Dendra2 under control of the *BAS4* promoter [O-137; pCK1244]
CKF1996	Figures 2B, 2C, and 3A; Supplemental Figure 2A and 2B	Transformant expressing both a fusion of the Bas4 signal peptide fused to EGFP under control of the *BAS4* promoter and a cytoplasmic tdTomato under control of the constitutive promoter of the *M. oryzae* ribosomal protein 27 [CKF315; pCK1292]
CKF2180[a]	Supplemental Figure 1	Transformant expressing the Bas4 signal peptide fused to EGFP under control of the *BAS4* promoter [O-137; pBV324]
CKF2187	Figures 4B, 4C, and 6A; Supplemental Figures 3B, 3C, 3D, and 4	Transformant expressing both a fusion of the Bas4 signal peptide fused to EGFP under control of the *BAS4* promoter and a fusion of histone H1 with tdTomato under control of the constitutive promoter P27 [O-137; pCK1312]
CKF3267	Supplemental Figure 2C	Transformant expressing the Bas4 signal peptide fused to mCherry under control of the *BAS4* promoter [O-137; pCK1594]

[a]Two independent transformants containing the same construct showing consistent fluorescence patterns

[b]*M. oryzae* strain O-137 was isolated from rice (*Oryza sativa*) in China (Orbach, et al. 2000).
