# Supplementary material for: Disruption of the Interfacial Membrane Leads to Magnaporthe oryzae Effector Re-location and Lifestyle Switch During Rice Blast Disease
Source: Front Cell Dev Biol. 2021 Jun 17;9:681734. doi: 10.3389/fcell.2021.681734 (PMC8248803; doi:10.3389/fcell.2021.681734)
Supplement: Supplementary Table 2 — Key plasmids used in this study. [file Table_2.pdf]

Supplementary Table 2. Key Plasmids Used in This Study

Clone	Description
pBV324	1 kb *Eco*RI-*Bam*HI fragment containing the *BAS4* promoter and 72 bp *Bam*HI-*Pst*I fragment encoding the Bas4 signal peptide cloned in the *Eco*RI and *Pst*I sites of pBV176 (C-terminal translational fusion of EGFP to the Bas4 signal peptide); Khang et al., 2010
pBV591	P_{PWL2}:PWL2CDS:mCherry:NLS:Nopaline synthase gene (Nos) terminator and P_{BAS4}:BAS4CDS:EGFP:Ter cloned in *Eco*RI and *Hind*III sites of pBHt2; Khang et al., 2010
pCK1244	1.3 kb *Eco*RI-*Bam*HI fragment containing the *BAS4* promoter and coding sequence from pBV587, 0.7 kb *Bam*HI-*Bsr*GI fragment containing Dendra2 from pCK1206, and 0.3 kb *Bsr*GI-*Sal*I fragment containing the Nos terminator from pBV360 (same as pAN583; Nelson et al., 2007) cloned into *Eco*RI-*Sal*I sites of pBHt2
pCK1292	0.5 kb *Eco*RI-*Bam*HI fragment containing the P27 promoter from pBV167 and 1.7 kb *Bam*HI-*Hind*III fragment containing tdTomato from pBV359 (same as pAN582; Nelson et al., 2007) cloned into *Eco*RI-*Hind*III sites of pBGt
pCK1312	2.2 kb *Eco*RI-*Bam*HI fragment of pBV229 containing the P27 promoter and histone H1 and 1.7 kb *Bam*HI-*Sal*I fragment of pBV359 containing tdTomato cloned into *Eco*RI-*Xho*I sites of pBV324
pCK1594	1 kb *Eco*RI-*Bam*HI fragment containing the *BAS4* promoter and sequence encoding the signal peptide from pBV722 and 1 kb *Bam*HI-*Hind*III fragment of containing mCherry:Nos terminator cloned into *Eco*RI-*Hind*III sites of pBGt
